# Highly sensitive electrochemical detection of hazardous 2,4-dinitrophenylhydrazine using MgCo-TiO_2_/g-C_3_N_4_ heterostructure nanocomposites

**DOI:** 10.1039/d5ra07106b

**Published:** 2025-11-24

**Authors:** Samuel Chufamo Jikamo, T. Siva Rao, P. Shyamala, Singupilla Sai Supriya, Sandhya Rani Nayak, Nageswararao Kadiyala, Winni Teja Dokka, M. Ravichandra, M. V. Kishore

**Affiliations:** a Dept of Chemistry, Andhra University Visakhapatnam 530003 India sivaraoau@gmail.com; b Dept of Chemistry, College of Natural and Computational Sciences, Wolaiat Sodo University Wolaita Sodo Ethiopia; c Dept of Chemistry, Dr V S Krishna Govt. Degree College (A) Visakhapatnam 530013 India; d Dept of Chemistry, Govt. Degree College Chintalapudi West Godavari 534460 India; e Indian Institute of Technology Patna C4-101, Staff Quarters Bihta Bihar 801106 India

## Abstract

In this study, MgCo-TiO_2_/g-C_3_N_4_ heterostructure nanocomposites were successfully synthesized for the electrochemical detection of 2,4-dinitrophenylhydrazine. The MgCo-TiO_2_/C_3_N_4_ heterostructure nanocomposites were synthesized by preparing bimetal (Mg–Co)-doped TiO_2_*via* a microwave-assisted sol gel method, followed by a thermal approach to coat it onto g-C_3_N_4_ nanosheets. The morphology, structure, composition and optical and electrochemical properties of the fabricated heterostructure nanocomposites were characterized by various analytical techniques including PXRD, UV-DRS, VB-XPS, PL, HRTEM, FESEM-EDX, FT-IR, EIS and CV. To develop an electrochemical sensor for 2,4-dinitrophenylhydrazine (2,4-DNPH), the MgCo-TiO_2_/g-C_3_N_4_ heterostructure nanocomposites were coated onto a glassy carbon electrode (GCE) by a drop-casting method. MgCo-TiO_2_/g-C_3_N_4_/GCE showed an excellent electroanalytical response for the oxidation of 2,4-DNPH in a lower pH environment. MgCo-TiO_2_/g-C_3_N_4_/GCE exhibited excellent selectivity, sensitivity (589.13 µA µM^−1^ cm^−2^), and a lower limit of detection (0.06 µM) in a wide linear range (0.1–0.9 µM) under optimized conditions. Furthermore, the sensor exhibited high repeatability (RSD 1.38%), reproducibility (RSD 3.57%), stability and recovery in real sample analysis.

## Abstract 2

In this study, MgCo-TiO_2_/g-C_3_N_4_ heterostructure nanocomposites were successfully synthesized for the electrochemical detection of 2,4-dinitrophenylhydrazine.

## Graphical abstract

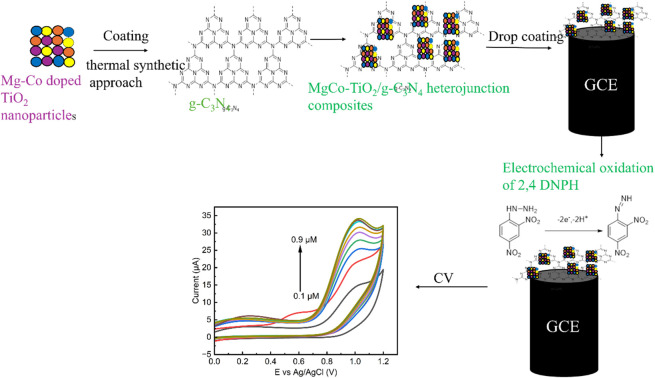

## 1. Introduction

The widespread industrial use of aromatic compounds has led to their increasing environmental emissions, raising serious concerns due to their toxicity, persistence, and bioaccumulation. Among these compounds, 2,4-dinitrophenylhydrazine (2,4-DNPH) is an aromatic compound containing both nitro and hydrazine functional groups. It is widely used as a raw material in the industrial production of explosives, pharmaceuticals, pesticides, metal plating agents, blowing agents, and photographic materials.^1,2^ In laboratories, 2,4-DNPH is commonly employed in analytical chemistry for the detection of aldehyde and ketone carbonyl groups through derivatization reactions^3,4^ as well as in the synthesis of aryl hydrazones with potential antifungal activity.^5^ 2,4-DNPH is an environmentally hazardous and poorly degradable compound because of its stable nitro and toxic hydrazine groups. Its major source of environmental contamination is untreated industrial and laboratory effluents, which can penetrate soil and groundwater^2^ or enter surface water bodies through erosion and runoff. Long-term exposure to 2,4-DNPH can lead to hepatotoxicity, skin disorders, central nervous system impairments, cardiovascular complications, and carcinogenic and mutagenic risks in humans.^6,7^

Therefore, the accurate detection and continuous monitoring of 2,4-DNPH in environmental matrices are essential to assess its levels and ecological risks. However, only a limited number of detection methods have been reported to date, including spectrometry,^8,9^ potentiometric titration,^10^ gas chromatography-mass spectrometry,^11^ chemiluminescence,^12^ and fluorescence sensing.^13^ Furthermore, these techniques are often expensive, non-portable, reagent-intensive, and time-consuming for real-sample analysis. In recent years, electroanalytical approaches using nanomaterial-modified electrode surfaces have gained increasing attention for the detection of 2,4-dinitrophenylhydrazine. The reported examples include poly-*para*-aminobenzoic acid–manganese oxide (P-*p*ABA-MnO_2_) composite-modified glassy carbon electrodes (GCEs),^2^ (P_8_W_48_/PDDA)_7_-modified ITO electrodes,^14^ PDDA/(Cu_4_P_4_W_30_/PDDAGO)_*n*_ assemblies,^15^ Zn,N-doped γ-cyclodextrin/GCE,^16^ and m-TiO_2_/FeTiO_3_@NCF/GCE.^17^ These sensors demonstrate excellent electroanalytical performances with low limits of detection, high electron transfer rates, sensitivity, selectivity, wide linear concentration ranges, and good reproducibility, primarily due to the synergistic effects of the composite materials on the modified electrode surface.

In electrochemical sensors, modifying the electrode surface with carbon-based catalyst materials provides significant advantages by forming conductive interfaces that enhance their interactions with the target analyte. For instance, reduced graphene oxide (rGO) provides excellent electrical conductivity and a large surface area,^18^ while graphene oxide (GO) is valued for its high chemical stability, large surface area, and abundant electrochemically active sites.^19^ Similarly, graphitic carbon nitride (g-C_3_N_4_) exhibits a high surface area, rapid electron transfer, and strong chemical stability.^20^ Among these materials, g-C_3_N_4_ has attracted considerable research interest owing to its simple preparation, medium band gap, low cost, wide availability, and chemical inertness.^21–23^ Furthermore, the presence of C–N bonds contribute improved catalytic electron transfer compared to other carbon-based materials due to the strong electronegativity and lone pair electrons on the nitrogen atoms, which offer abundant electrochemically active sites, enhance the electrode-analyte interactions, and facilitate rapid electron transfer through their conjugated structure.^24^ Several studies have reported the modification of electrodes with g-C_3_N_4_ nanosheets for electrochemical detection using voltammetric techniques, including the detection of hydrogen peroxide,^25^ oxalic acid,^26^ and tryptophan.^23^

However, g-C_3_N_4_ suffers from a relatively small surface area and rapid electron–hole recombination, which limit its electrocatalytic performance in electrochemical detection. Thus, to overcome these drawbacks, many researchers have focused on enhancing its surface area and electrical conductivity by forming heterostructures with various semiconductors. For instance, Co_3_O_4_ anchored g-C_3_N_4_ heterostructures exhibit improved electrical conductivity;^24^ an g-C_3_N_4_/CuWO_4_ nanocomposite-modified electrode exhibits excellent selectivity sensitivity for nitrofurazone detection;^27^ a Pd/CeO_2_/g-C_3_N_4_ nanocomposite demonstrates reduced electron–hole recombination and enhanced photocatalytic activity;^28^ g-C_3_N_4_Ag_2_ZrO_3_ composites show a high surface area and superior ethanol gas sensing ability;^29^ ZnO-modified g-C_3_N_4_ displays an enlarged specific surface area, narrowed band gap, and superior photocatalytic performance compared to pure g-C_3_N_4_;^30^ and a g-C_3_N_4_/MnO_2_/ZnO-modified GCE achieves faster electron transfer with greatly enhanced electrochemical sensing of metronidazole.^31^ Coupling g-C_3_N_4_ with transition metal oxide semiconductors noticeably enhances its surface area and electrical conductivity. Among these semiconductors, TiO_2_ exhibits superior physicochemical properties such as high stability, large surface area, porosity, strong catalytic activity, efficient electron transfer, and enhanced hydroxyl radical adsorption capacity, which collectively improve the electrochemical detection of organic compounds by increasing the electrode surface roughness.^32–34^

Nevertheless, TiO_2_ nanoparticles possess a wide band gap (3.2 eV), which leads to reduced electrical conductivity due to rapid electron–hole recombination. Thus, to address this limitation, researchers have explored doping TiO_2_ with metals and nonmetals to narrow its band gap without altering its crystallographic structure. The choice of synthesis method and dopant concentration plays a crucial role in determining the particle size, surface area, band gap, and electrical conductivity of TiO_2_. In our previous work, Ce–Ni co-doped TiO_2_ synthesized *via* an Aloe vera gel-mediated sol–gel method exhibited a small particle size, high surface area, reduced band gap, and enhanced photocatalytic activity for binary dye degradation.^35^ Furthermore, P. E. Imoisili and T. C. Jen reported that V-doped TiO_2_ prepared using a microwave-assisted method showed improved particle quality, surface area, uniform particle distribution and a narrowed band gap,^36^ which is attributed to its rapid nucleation and crystallization in a short irradiation time under microwave irradiation.^37^

In this work, a synergistic, highly sensitive, selective, and stable electrochemical sensor based on MgCo-TiO_2_/g-C_3_N_4_ heterostructure nanocomposites was developed for the detection of 2,4-dinitrophenylhydrazine (2,4-DNPH). Prior to the coupling of the MgCo-TiO_2_ and g-C_3_N_4_ materials, TiO_2_ was doped with Mg and Co *via* a microwave-assisted sol gel method with systematically optimized dopant concentrations and microwave powers to narrow its band gap and enhance its conductivity by promoting efficient charge separation. Integration of the TiO_2_ nanoparticles with g-C_3_N_4_ nanosheets resulted in the formation of MgCo-TiO_2_/g-C_3_N_4_ heterostructure nanocomposites. In this system, g-C_3_N_4_ provided strong interactions with 2,4-DNPH through hydrogen bonding and π–π stacking, while the MgCo-doped TiO_2_ nanoparticles enhanced the conductivity of g-C_3_N_4_ by increasing its surface area, narrowing its band gap, and suppressing electron–hole recombination. The synergistic coupling of both materials significantly improved the electrochemical detection performance toward 2,4-DNPH. The fabricated MgCo-TiO_2_/g-C_3_N_4_/GCE sensor demonstrated high selectivity, sensitivity, and stability toward the detection of 2,4-DNPH, emphasizing both the novelty and practical relevance of our approach.

## 2. Experiment

### 2.1. Chemicals and reagents

The precursors used for the synthesizing magnesium (Mg^2+^)- and cobalt (Co^2+^)-doped titanium oxide nanoparticles coated with graphitic carbon nitride (g-C_3_N_4_) were magnesium nitrate hydrate (Mg(NO_3_)_2_·6H_2_O, 99%), cobalt nitrate hexahydrate (Co(NO_3_)_2_·6H_2_O, 98%), tetra-*n*-butyl orthotitanate (Ti(OBu)_4_, 98%) and urea (NH_2_CONH_2_). 2,4-Dinitrophenylhydrazine (97%) was used for electrocatalytic detection applications. All chemicals were procured from Sigma-Aldrich. Ethanol (C_2_H_5_OH, 100%) from Hayman (UK) served as the solvent during the synthesis, while nitric acid (HNO_3_) from Merck (Germany) was used for surface modification during the synthesis of the TiO_2_ nanoparticles. All chemicals were analytical reagent (AR) grade and used without further purification.

### 2.2. Synthesis of MgCo co-doped TiO_2_ nanoparticles

The MgCo co-doped TiO_2_ nanoparticles were synthesized *via* the microwave-assisted sol gel method in two steps. Firstly, dopants with five different weight percentages were synthesized *via* the sol gel method. Among them, the one with the smallest crystalline particle size and lowest band gap was selected and further subjected to microwave irradiation to further decrease the crystalline particle size and band gap, while enhancing the specific surface area of MgCo-TiO_2_.

#### 2.2.1 .Using sol gel method

To optimize the dopant weight percentage, five different weight percentages of each dopant ratio to titanium was synthesized using the sol–gel method. Solution 1 was prepared by mixing 20 mL of tetra-*n*-butyl orthotitanate [Ti(OBu)_4_] with 40 mL of ethanol in a 250 mL Pyrex beaker. Ethanol enhanced the miscibility between the organic and aqueous phases, facilitating better interaction of Ti(OBu)_4_ with water. After stirring the mixture for 30 min, 3.2 mL of HNO_3_ was added under continuous stirring to catalyze and control the hydrolysis and condensation reactions, thus stabilizing the sol formation. The second dopant solution was prepared by dissolving 0.25 wt% (0.073 g) of Mg(NO_3_)_2_·6H_2_O and 0.75 wt% (0.0475 g) of (Co(NO_3_)_2_·6H_2_O) in a separate 250 mL Pyrex glass beaker containing 40 mL of ethanol and 7.2 mL of Milli-Q water with continuous stirring. After stirring solution 1 for 1 h, solution 2 was added dropwise into solution 1, and the mixture was stirred for an additional one hour until a sol was formed. The sol was kept in the dark for 48 h to allow gel formation. Subsequently, the gel was dried in a hot oven at 72 °C for 24 h to obtain controlled solvent removal and structural integrity before high-temperature treatment, which was ground repeatedly to obtain a fine powder. The resulting fine powder was calcined in a muffle furnace at 450 °C for 5 h to promote crystallization into the anatase TiO_2_ phase and remove residual organic components, yielding pure and finely structured TiO_2_ nanoparticles. The calcined sample was collected, cooled, ground and labeled as MgCo-TiO_2_ nanoparticles (MCT1). Using the same procedure, other samples with varying dopant weight percentages of 0.5 wt% Mg and 0.5 wt% Co (MCT2), 0.75 wt% Mg and 0.25 wt% Co (MCT3), 1 wt% Mg and 0.25 wt% Co (MCT4), and 0.25 wt% Mg and 1 wt% Co (MCT5) were prepared. Bare TiO_2_ was also prepared using the same procedure without adding dopants.

#### 2.2.2 .By microwave-assisted sol gel method

**Scheme 1:**
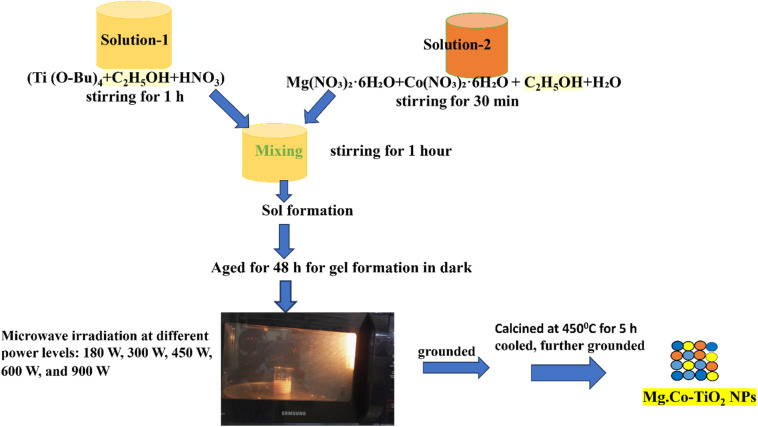
Schematic of the microwave-assisted sol gel synthesis of MgCo-TiO_2_ NPs.

Based on PXRD and UV-Vis DRS characterization, the sample with 0.25 wt% Mg and 1 wt% Co (MCT5) exhibited the smallest crystallite size (7.28 nm) and the lowest band gap (2.67 eV) among the five synthesized samples (MCT1, MCT2, MCT3, MCT4 and MCT5). To further reduce the crystallite size and band gap, while enhancing the specific surface area of the MgCo-TiO_2_ nanoparticles, using the MCT5 composition, the synthesis was carried out to the sol stage and divided into five equal portions, each placed in a 150 mL Pyrex beaker. The sols were kept in the dark for 48 h to form gels, and then irradiated at 180, 300, 450, 600, and 900 W, with the corresponding irradiation times of 20, 8, 7, 3, and 2.5 min (Table 1), producing samples labeled MCT5M1, MCT5M2, MCT5M3, MCT5M4 and MCT5M5, respectively. All samples were subsequently collected, ground, calcined at 450 °C for 5 h, cooled, and reground for further characterization (Scheme 1). The microwave-assisted method yielded higher-quality products with smaller crystallites with a uniform particle distribution.

**Table 1:** Power level, irradiation time and labelled names for catalysts

Power level	Output	Irradiation time (min)	Catalyst name
Low	180 W	20	MCT5M1
Medium low	300 W	8	MCT5M2
Medium	450 W	7	MCT5M3
Medium high	600 W	3	MCT5M4
High	900 W	2.5	MCT5M5

### 2.3. Fabrication of MgCo-TiO_2_/g-C_3_N_4_ heterostructure composites

Using the optimized TiO_2_ sample (MCT5M3), prepared with 0.25 wt% Mg and 1 wt% Co dopants under 450 W irradiation power, MgCo-TiO_2_/g-C_3_N_4_ heterostructure composites were synthesized following a reported method with minor modifications.^38^ In a typical procedure, 5 g of urea was placed in a crucible and heated in a muffle furnace at 600 °C for 3 h, then allowed to cool, and finely ground, obtaining a yellow nanosheet powder. A 1 : 3 mass ratio of MgCo-TiO_2_ to g-C_3_N_4_ was dispersed in 20 mL of distilled water by continuous stirring, followed by ultrasonication for 30 min to achieve a uniform dispersion. The resulting suspension was transferred into a ceramic crucible and heated at 72 °C for 6 h. The product was cooled, ground, and further calcined at 400 °C for 2 h in a muffle furnace. After cooling, the material was finely ground again and collected as MgCo-TiO_2_/g-C_3_N_4_ heterostructure composites.

### 2.4. Preparation of MgCo-TiO_2_/g-C_3_N_4_-modified glassy carbon electrode

Prior to modification, a bare glassy carbon (GC) electrode was sequentially polished with 1.0, 0.3, and 0.05 µm alumina powders, rinsed with distilled water, ultrasonicated in ethanol and deionized water, and finally dried at room temperature. For electrode modification, 2 mg of MgCo-TiO_2_/g-C_3_N_4_ was dispersed in 2 mL of ethanol and ultrasonicated for 10 min to obtain a uniform suspension. A 5 µL aliquot of the suspension without binder was drop-casted onto the pretreated GC electrode surface and air-dried for 30 min prior to use. Three identical electrodes were prepared following the same procedure for reproducibility evaluation. The actual loading on GCE was calculated using the suspension concentration (2 mg/2 mL = 1 mg/mL), volume drop-cast (5 µL = 0.005 mL), and GCE diameter (3 mm = 0.3 cm), corresponding to a geometric area of 0.07 cm^2^. Based on these parameters, the calculated loading on the GCE electrode was approximately 70.7 µg cm^−2^.

### 2.5. Characterization instruments

The crystallite sizes of the fabricated catalysts were determined based on FWHM and diffraction angle data using PXRD (Ultima IV Rigaku, 40 kV/30 mA) with Cu Kα radiation, scanned at 2° min^−1^ over the 2*θ* range of 2–80° at room temperature. Optical band gaps and absorption edges were measured using a Shimadzu 3600 UV-Vis DRS NIR spectrophotometer in the range of 200–800 nm with BaSO_4_ as a reference. Elemental composition and surface morphology were examined by energy-dispersive X-ray spectroscopy (EDX) coupled with field-emission SEM (FESEM) operating at 20 kV. High-resolution transmission electron microscopy (HRTEM) was employed to analyze the surface morphology and nanostructure of the heterostructure composites. The surface area, pore size, pore volume, and pore distribution of the synthesized catalysts were analyzed using a BET surface area analyzer (Gemini VII 2390, Micromeritics). X-ray photoelectron spectroscopy (XPS) was employed to determine the oxidation states, elemental composition, and binding energies. The functional groups in the metal oxides were characterized by FTIR (Bruker Hyperion 3000 with Vertex 80) in the range of 400–4000 cm^−1^. Photoluminescence (PL) spectroscopy was carried out using a Horiba Jobin Yvon FluoroMax-4 spectrofluorometer equipped with a PMT (50 V) to study electron–hole recombination. Excitation and emission monochromators were set at 2.5 nm slit widths, and samples were measured in a four-sided quartz cuvette suitable for excitation and emission detection. The electrochemical behavior of the catalyst-modified electrodes was evaluated using cyclic voltammetry (CV), electrochemical impedance spectroscopy (EIS), chronocoulometry, and linear sweep voltammetry (LSV). All measurements were performed on a CHI6005E electrochemical analyzer (CHI Inc., USA) in a three-electrode system, employing a glassy carbon electrode (GCE) as the working electrode, platinum wire as the counter electrode, and Ag/AgCl as the reference electrode.

### 2.6. Optimization and electrochemical detection of 2,4-dinitrophenylhydrazine

The optimization was performed by evaluating the effects of pH, 2,4-dinitrophenylhydrazine concentration, and scan rate on the electrochemical response of a MgCo-TiO_2_/g-C_3_N_4_-modified GCE using the cyclic voltammetry technique. Experiments were conducted in 0.1 M phosphate buffer solution containing 0.1 M KCl as the supporting electrolyte. The pH of the buffer was varied from 3.0 to 11.0, while the concentration of 2,4-dinitrophenylhydrazine ranged from 0.1 µM to 0.9 µM. The scan rate effect was tested in the range of 20 mV s^−1^ to 90 mV s^−1^. The detection of 2,4-dinitrophenylhydrazine was carried out using the linear sweep voltammetry (LSV) technique across a range of concentrations from 0.1 µM to 0.9 µM (0.1, 0.2, 0.3, 0.4, 0.5, 0.6, 0.7, 0.8 and 0.9 µM).

## 3. Results

### 3.1. Structural, morphological and optical characterization

#### 3.1.1. XRD analysis

**Fig. 1:**
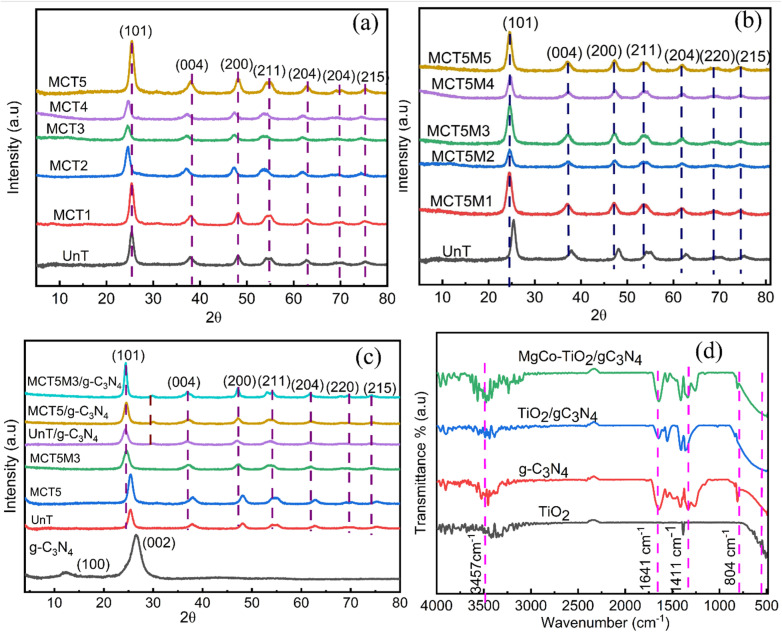
PXRD patterns of sol gel-synthesized particles (a), microwave-assisted sol gel synthesized at different power levels (b) and g-C_3_N_4_ and coated heterostructure composites (c). FT-IR spectra of TiO_2_, g-C_3_N_4_, TiO_2_/g-C_3_N_4_ and MgCo-TiO_2_/g-C_3_N_4_ (d).

The crystalline phases and composition of the prepared samples of undoped (pure TiO_2_), five different percentage weight ratios of Mg to Co co-doped TiO_2_ nanoparticles, which were synthesized *via* the sol–gel and microwave-assisted sol–gel methods, g-C_3_N_4_ and Mg-Co-TiO_2_/g-C_3_N_4_ heterostructure composites were characterized by PXRD. The PXRD peaks of the undoped TiO_2_ nanoparticles were observed at 2*θ* values of 25.43°, 37°, 48°, 54.32°, 62.6°, 69.72°, and 75.5°, corresponding to the anatase phase diffraction planes of (101), (004), (200), (105), (204), (220), and (215), respectively. After Mg and Co co-doping at various weight ratios *via* the sol–gel method, no new peaks appeared; however, the slight shift in the main peak from 25.43° to 24.58° indicated the successful incorporation of the dopant into the TiO_2_ lattice (Fig. 1a). All the synthesized samples remained in the anatase phase, as the low dopant concentrations did not alter their crystallographic structure. Similarly, in the microwave-assisted sol gel method (Fig. 1b), no additional peaks were detected, though slight peak shifts were observed. The PXRD pattern of the synthesized g-C_3_N_4_ showed two characteristic peaks at 2*θ* = 12.82° (100), attributed to tri-*s*-triazine units, and 26.74° (002), corresponding to the conjugated aromatic structure. In the case of the MgCo-TiO_2_ particles coated with g-C_3_N_4_ synthesized *via* the microwave-assisted sol gel method, their diffraction peaks appeared at 2*θ* = 24.58°, 37°, 47.2°, 53.9°, 62°, 69.62°, and 74.77°, indexed to the anatase TiO_2_ planes of (101), (004), (200), (105), (204), (220), and (215), respectively (Fig. 1c).

The coupling of g-C_3_N_4_ with undoped TiO_2_ and MgCo-TiO_2_ did not significantly alter their anatase phase structure. However, a weak peak at 29.62° was observed in both TiO_2_/g-C_3_N_4_ and MgCo-TiO_2_/g-C_3_N_4_, while the peak for g-C_3_N_4_ at 12.82° disappeared and the peak at 26.74° shifted to 29.62°, likely due to interfacial interactions (Mg–O–N, Co–O–N, or Ti–O–N) in the heterostructure. The peaks for g-C_3_N_4_ were also masked by stronger anatase TiO_2_ signals due to the shielding effect.^39^ The crystallite sizes were calculated using the Debye–Scherrer equation (*D* = *kλ*/*β* cos *θ*, where *β* is the FWHM, *k* = 0.9, *θ* is the Bragg angle, and *λ* is the Cu Kα wavelength). As dopants were introduced in the TiO_2_ lattice, the crystallite size of TiO_2_ increased. Among the doped samples, the MCT5 sample with a higher Co content (lower Mg : Co ratio) allows larger Co^2+^ ions (0.745 Å) to substitute Ti^4+^ (0.605 Å), leading to lattice distortion and the formation of oxygen vacancies for charge compensation. The Co dopants act as heterogeneous nucleation centers, enhancing nucleation, while the moderate amount of Mg introduces additional defect states that inhibit grain growth. Consequently, the MCT5 sample exhibits the smallest crystallite size of 7.28 nm among the ratios.

Consequently, MCT5 was further subjected to microwave irradiation at different power levels to obtain smaller crystallite sizes and improved particle-quality anatase TiO_2_. Among them, the sample irradiated at 450 W showed the smallest crystallite size of 6.28 nm. Overall, Mg–Co co-doped TiO_2_ prepared by microwave synthesis exhibited finer particles and superior quality due to its rapid nucleation, shorter reaction times, and lower activation energy, which minimize side reactions and phase transitions. The average crystallite size of pristine g-C_3_N_4_ was 25.05 nm, which decreased to 11.39 nm when coated with pure TiO_2_ and further to 6.52 nm with MgCo-TiO_2_ (Table 2).

**Table 2:** UV-DRS band gap and PXRD crystalline particle size results

Catalyst name	Average crystalline size (nm)	Band gap (eV)	Catalyst name	Average crystalline size (nm)	Bandgap (eV)
UnT	5.62	3.06	MCT5M2	9.21	2.68
MCT1	38.05	2.72	MCT5M3	6.28	2.58
MCT2	17.23	2.74	MCT5M4	9.53	2.77
MCT3	18.53	2.84	MCT5M5	8.38	2.79
MCT4	7.38	2.70	g-C_3_N_4_	25.05	2.72
MCT5	7.28	2.67	TiO_2_/g-C_3_N_4_	11.39	2.84
MCT5M1	7.52	2.69	MgCo-TiO_2_/g-C_3_N_4_	6.52	2.41

#### 3.1.2. UV-visible DRS analysis

**Fig. 2:**
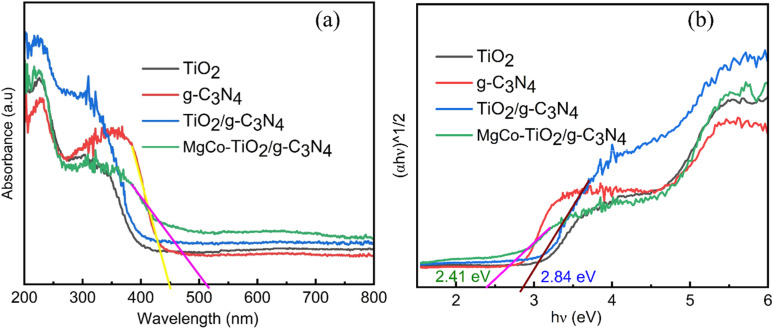
UV-Vis DRS absorbance spectra of TiO_2_, g-C_3_N_4_, TiO_2_/g-C_3_N_4_ and MgCo-TiO_2_/g-C_3_N_4_ (a) and square root of their Kubelka–Munk functions (b).

The optical properties of the synthesized samples were examined using UV-Vis DRS in the range of 200–800 nm. Undoped TiO_2_ (UnT) showed absorption only below 400 nm, confirming its UV-limited activity. In contrast, Mg–Co co-doped TiO_2_ exhibited absorption in both the UV and visible regions (Fig. S1a), which is attributed to its dopant-induced intermediate energy states and electron traps, which shifted its absorption edge into the visible region. Among the doped samples, 0.25% Mg and 1% Co (MCT5) displayed the strongest visible-light harvesting. Furthermore, microwave-assisted sol gel synthesized MCT5 at 450 W (MCT5M3) exhibited greater visible absorption than the other irradiation powers (180 W, 300 W, 450 W, 600 W, and 900 W) (Fig. S1c). Pristine g-C_3_N_4_ showed an absorption edge at 447.74 nm, indicating visible light activity, while coupling with MgCo-TiO_2_ further shifted the absorption peak. Overall, the MgCo-TiO_2_/g-C_3_N_4_ heterostructure nanocomposites demonstrated the highest visible light absorption compared to g-C_3_N_4_, TiO_2_, and TiO_2_/g-C_3_N_4_ (Fig. 2a).

The band gap (*E*_g_) of all the fabricated samples was calculated from their Tauc plots using the Kubelka–Munk equation: *α*ℏ*ν* = *A* (ℏ*ν* − *E*_g_)^*n*/2^, where *α* is the absorption coefficient, ℏ is Plank's constant, *ν* is the frequency of light, *E*_g_ is the band gap energy and *A* is a constant. All five percentages of Mg and Co-doped TiO_2_ nanoparticles synthesized *via* the sol gel method showed a lower band gap compared to undoped TiO_2_ (Fig. S1b). This reduction was observed due to the introduction of dopants atoms. Particularly, the 0.25% Mg and 1% Co-doped TiO_2_ (MCT5) showed a significant reduction in the band gap of undoped TiO_2_ by lowering it to 2.67 eV compared with other dopant concentration (Table 2). The higher concentration of Co dopant, with its d-orbitals might have a great interacting chance with TiO_2_ electronic state, reducing the band gap. The co-doping with Mg also enhances the formation of defect states and oxygen vacancies in the TiO_2_ lattice.

Microwave-assisted sol gel synthesis further reduced the band gap by promoting rapid nucleation, controlled crystallization, and suppression of side reactions, yielding uniform, high-quality nanoparticles. Among the samples, MCT5 irradiated at 450 W (MCT5M3) exhibited the lowest band gap of 2.58 eV (Fig. S1d). Pure g-C_3_N_4_ showed a band gap of 2.72 eV, consistent with reported values,^38^ confirming its successful synthesis. Coating TiO_2_ and MgCo-TiO_2_ onto g-C_3_N_4_ resulted in the formation of heterostructure nanocomposites with band gaps of 2.84 eV and 2.41 eV, respectively (Fig. 2d). In the MgCo-TiO_2_/g-C_3_N_4_ heterostructure nanocomposites, Mg and Co facilitated efficient charge transfer and separation, enhancing the electronic state overlap. Notably, the microwave-synthesized MgCo-TiO_2_/g-C_3_N_4_ exhibited the lowest band gap, suggesting its superior electrocatalytic potential.

#### 3.1.3. XPS

**Fig. 3:**
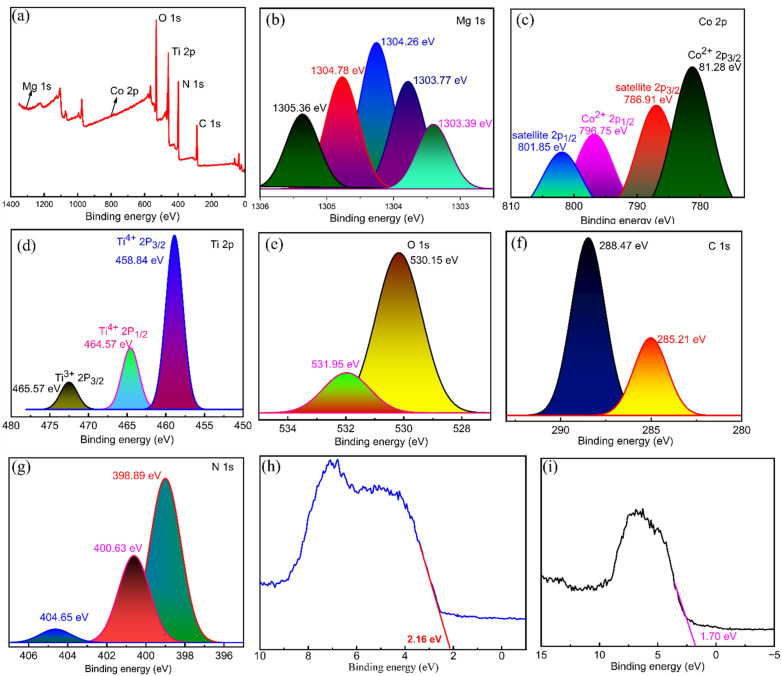
XPS survey spectrum of MgCo-TiO_2_/g-C_3_N_4_ (a), high-resolution spectra of Mg 1s (b), Co 2p (c), Ti 2p (d), O 1s (e), C 1s (f), and N 1s (g) and VBM of TiO_2_ (h) and MgCo-TiO_2_/g-C_3_N_4_ (i).

XPS analysis was conducted to investigate the chemical environments for each element in MgCo-TiO_2_/g-C_3_N_4_. As shown in Fig. 3a in the XPS survey, no impurities were seen and all the elements in the samples were present (Mg, Co, Ti, O, C, and N). The fitted peak components, corresponding areas, FWHM, and quantitative atomic% of each element are listed in Table 3. Each element was verified in its XPS spectrum including Mg 1s state, Co 2p state, Ti 2p state, O 1s state, C 1s state and N 1s state. The Mg 1s spectrum exhibited various binding energies, showing the multiple chemical environments of magnesium within the composites. The main intensity band at the binding energy of 1304.249 eV and the second peak at 1304.76 eV corresponded to the substitution of Mg^2+^ into the TiO_2_ lattice (Ti–O–Mg) by doping. The other two peaks at 1303.78 eV and 1303.4 eV are ascribed to the defect region with oxygen or interaction interface with g-C_3_N_4_ in the heterostructure composite (Fig. 3b). The main photoelectron Co 2p core-level peaks in Fig. 3c are observed at binding energies of 781.28 eV (Co 2p_3/2_) and 796.75 eV (Co 2p_1/2_). Two satellite peaks were observed at the binding energies of 786.91 eV and 801.85, corresponding to Co 2p_3/2_ and Co 2p_1/2_, respectively, further the confirming the presence of Co^2+^ in a high-spin configuration and indicating the presence of Co^2+^ in a Ti–O–Co or Co–O–N–C coordination environment. These results indicate that Co^2+^ ions were successfully incorporated into the TiO_2_ lattice by substituting Ti^4+^ ions. As shown in Fig. 3d, the Ti 2p core-level peaks observed at binding energies of 458.84 eV (Ti 2p_3/2_) and 464.57 eV (Ti 2p_1/2_) confirm the existence of Ti–O bonds within the TiO_2_ lattice. A third peak appearing at a binding energy of 465.57 eV, positioned above the main Ti 2p_3/2_ peak and close to the Ti 2p_1/2_ peak, is identified as a satellite peak. This feature likely arises from the influence of the Mg and Co dopants or from the interface interaction between Ti and g-C_3_N_4_ within the heterostructure composite. The O 1s core-level spectrum displays two distinct binding energies attributed to the presence of dopants and the formation of interface states in the heterostructure MgCo-TiO_2_/g-C_3_N_4_ composite (Fig. 3e). The peak at 530.15 eV corresponds to lattice oxygen, likely associated with Ti–O–Ti, Ti–O–Mg, or Ti–O–Co bonds. The second peak at 531.98 eV is attributed to oxygen vacancies or defects, most likely resulting from doping or the formation of O–C or O–N bonds. The C 1s core-level spectrum shows two binding energy peaks (Fig. 3f), *i.e.*, the main peak at a binding energy of 288.47 eV, which corresponds to sp^2^-hybridized carbon (C=N–C), and at 285.21 eV, revealing the C–C or C=C bond conjugated aromatic structure of the g-C_3_N_4_ composite. These findings confirm that the g-C_3_N_4_ composite was successfully synthesized. As illustrated in Fig. 3g, the N 1s core-level spectrum reveals three distinct binding energy peaks, indicating the influence of the dopants in the MgCo-TiO_2_/g-C_3_N_4_ composite. The first peak at 398.89 eV corresponds to electron-rich nitrogen from sp^2^-hybridized nitrogen (C=N–C) in the g-C_3_N_4_ structure. The second peak at 400.63 eV is likely due to electron donation from graphitic nitrogen to the π-conjugated system. The third peak observed at 404.65 eV is attributed to the formation of Mg–N, Ti–N or Co–N bonding interface interactions in the heterostructure environment. The fitted peak components, corresponding areas, FWHM values, and quantitative atomic percentages of each element are summarized in Table 3.

**Table 3:** XPS fitted peak components, corresponding areas, FWHM values, and quantitative atomic percentages

Name of element	Peak BE (eV)	FWHM (eV)	Area (P) CPS (eV)	Atomic (%)
Ti 2p	459	3.15	1 562 284.71	12.07
O 1s	530.87	2.94	1 318 786.63	24.17
N 1s	399.44	3.33	1 127 019.2	32.2
C 1s	288.18	4.35	695 985.5	30.93
Co 2p	781.71	3.32	85 454.96	0.31
Mg 1s	1303.78	2.32	29 930.69	0.32

#### 3.1.4. VB-XPS

**Scheme 2:**
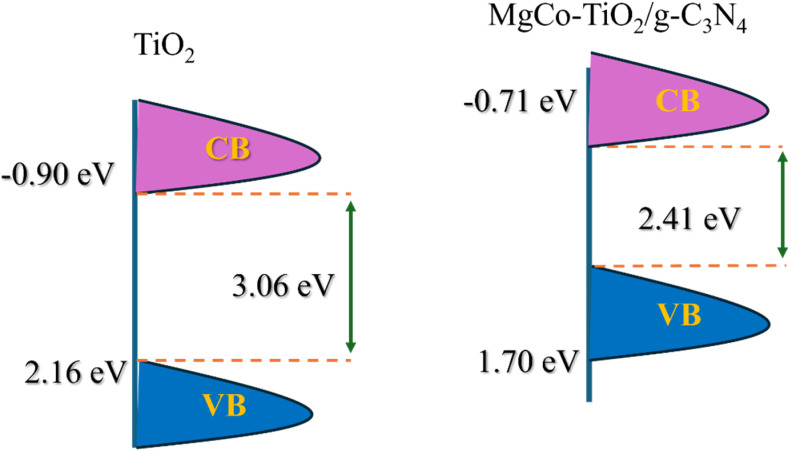
Schematic for the VB and CB shifting during the coupling of MgCo-TiO_2_ and g-C_3_N_4_.

The valence binding maximum (VBM) was determined by extrapolating the peak tangent line with the baseline for the undoped TiO_2_ and MgCo-TiO_2_/g-C_3_N_4_ heterostructure nanocomposites. The valence band maximum (VBM) of the undoped TiO_2_ nanoparticles was located at 2.16 eV below the zero potential energy level (Fig. 3h). Upon co-doping TiO_2_ with magnesium and cobalt *via* the microwave-assisted sol gel method, and subsequently coating it onto g-C_3_N_4_ nanosheets, the valence band edge shifted to 1.70 eV (Fig. 3i), indicating a 0.46 eV upward movement of VB toward the Fermi level compared to undoped TiO_2_. This enhanced visible-light absorption improves photon utilization and promotes the generation of more photo-carriers under visible light. The introduction of Mg^2+^/Co^2+^ dopant states and coupling with g-C_3_N_4_ creates mid-gap states and forms a heterojunction, providing efficient charge transfer pathways where electrons and holes are spatially separated or trapped in different sites. The conduction band (CB) positions of undoped TiO_2_ and the MgCo-TiO_2_/g-C_3_N_4_ heterostructure were estimated using the relation *E*_g_ = *E*_VB_ − *E*_CB_, with the values of −0.90 eV and −0.71 eV, respectively. The corresponding schematic representation is shown below for clarity (Scheme 2). The undoped TiO_2_ exhibits a more negative conduction band potential than the MgCo-TiO_2_/g-C_3_N_4_ heterostructure; consequently, the photogenerated electrons transfer from TiO_2_ to the MgCo-TiO_2_/g-C_3_N_4_ composite.

#### 3.1.5. Photoluminescence (PL) spectral study

Photoluminescence (PL) analysis was performed to evaluate the electron–hole separation efficiency of the synthesized semiconductors and heterostructure composites. As shown in Fig. 7d, pure g-C_3_N_4_ exhibited strong PL emission, indicating the rapid recombination of electron–hole pairs. Coating the g-C_3_N_4_ nanosheets with undoped TiO_2_ reduced the emission intensity, reflecting suppressed recombination due to the formation of a heterostructure and electron transfer from TiO_2_ to g-C_3_N_4_, as TiO_2_ possesses a higher conduction band. Mg and Co doping in TiO_2_ introduced trap states, which further promoted charge transfer, minimized recombination, and enhanced the redox activity. Consequently, MgCo-TiO_2_/g-C_3_N_4_ exhibited the lowest PL intensity, signifying the most effective recombination suppression and improved conductivity. Overall, the PL emission intensity decreased in the order of g-C_3_N_4_ > TiO_2_/g-C_3_N_4_ > MgCo-TiO_2_/g-C_3_N_4_, while the conductivity increased in the same sequence, confirming that MgCo-TiO_2_/g-C_3_N_4_ is a highly promising electrocatalyst for facilitating electron transfer between the electrode and electrolyte.

#### 3.1.6. Surface area and pore distribution study (BET) analysis

**Fig. 4:**
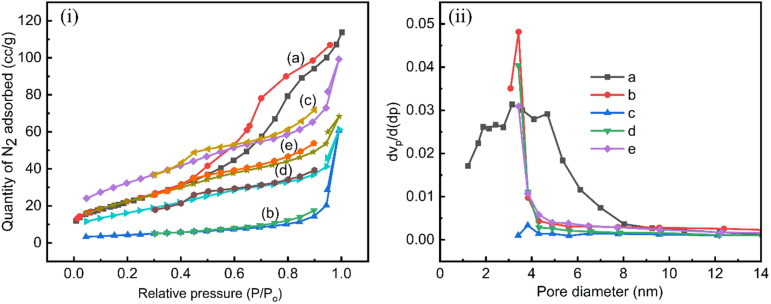
N_2_ adsorption–desorption isotherms (i) and BJH pore size distribution of (ii) TiO_2_ (a), g-C_3_N_4_ (b), MgCo-TiO_2_ (c), TiO_2_/g-C_3_N_4_ (d) and MgCo-TiO_2_/g-C_3_N_4_ (e).

The surface area and pore distribution of the synthesized materials were characterized using nitrogen gas adsorption–desorption at 77 K based on the Brunauer–Emmett–Teller (BET) surface area analysis method. The N_2_ adsorption–desorption isotherms of all the synthesized materials, as shown in Fig. 4a, exhibit hysteresis loops characteristic of the H3 type, with the loops appearing at higher relative pressures (*P*/*P*_o_) ranging from 0.4 to 1.0 except for g-C_3_N_4_, which shows a shift between 0.7 and 1.0. Additionally, the presence of two capillary condensation steps indicates type IV isotherms, according to the IUPAC classification. When undoped TiO_2_ and Mg–Co co-doped TiO_2_ (synthesized *via* the microwave-assisted method) were coated onto the surface of g-C_3_N_4_, the specific surface area of the resulting heterostructure composites increased compared to that of pure g-C_3_N_4_. The obtained BET specific surface area of TiO_2_, g-C_3_N_4_, MgCo-TiO_2_, TiO_2_/g-C_3_N_4_ and MgCo-TiO_2_/g-C_3_N_4_ was observed to be 83.46 m^2^ g^−1^, 15.47 m^2^ g^−1^, 116.06 m^2^ g^−1^, 59.63 m^2^ g^−1^ and 81.61 m^2^ g^−1^, respectively (Table 4). g-C_3_N_4_ has a narrow band gap, is visible light active and has more electron-rich species than TiO_2_ but it has low porosity and limited surface area. For instance, in a reported study,^38^ TiO_2_ was combined with g-C_3_N_4_, leading to an increase in the heterostructure surface area from 29.4 m^2^ g^−1^ to 36.2 m^2^ g^−1^. Similarly, in another reported study,^40^ copper and chromium-doped g-C_3_N_4_ achieved a surface area of 15.7 m^2^ g^−1^. Furthermore, when the surface of g-C_3_N_4_ was modified with green-synthesized TiO_2_, its BET surface area increased from 10.41 m^2^ g^−1^ to 32.52 m^2^ g^−1^, which enhanced the photocatalytic performance in a reported study.^41^

**Table 4:** Specific surface area and total pore volume and average pore diameter of the particles

Catalyst	*S*_BET_ (m^2^ g^−1^)	Total pore volume (cm^3^ g^−1^)	Average pore width (nm)
TiO_2_	83.46	0.17	3.15
g-C_3_N_4_	15.47	0.09	3.83
MgCo-TiO_2_	116.06	0.12	3.43
TiO_2_/g-C_3_N_4_	59.63	0.08	3.41
MgCo-TiO_2_/g-C_3_N_4_	81.61	0.07	3.82

These reported works show the significant impact of the surface modification of g-C_3_N_4_ on its photocatalytic and adsorption capacity. In this work, surface modification of g-C_3_N_4_ with undoped TiO_2_ and Mg–Co doped TiO_2_ increased the heterostructure surface areas to 59.63 m^2^ g^−1^ and 81.61 m^2^ g^−1^, respectively, which are significantly higher than previously reported values. This improvement is attributed to the microwave-assisted method and the dopants, which generated smaller TiO_2_ crystallites, increased particle numbers, and inhibited excessive crystal growth. Furthermore, the pore volume and pore diameter of TiO_2_, g-C_3_N_4_, MgCo-TiO_2_, TiO_2_/g-C_3_N_4_, and MgCo-TiO_2_/g-C_3_N_4_ were determined using the Barrett–Joyner–Halenda (BJH) method, as shown in Fig. 4b. The pore volume and pore average diameter of TiO_2_, g-C_3_N_4_, MgCo-TiO_2_, TiO_2_/g-C_3_N_4_ and MgCo-TiO_2_/g-C_3_N_4_ are 0.17 cm^3^ g^−1^, 0.09 cm^3^ g^−1^, 0.12 cm^3^ g^−1^, 0.08 cm^3^ g^−1^ and 0.07 cm^3^ g^−1^ and 3.15 nm, 3.83 nm, 3.43 nm, 3.41 and 3.82 nm, respectively.

#### 3.1.7. Surface and morphology study

**Fig. 5:**
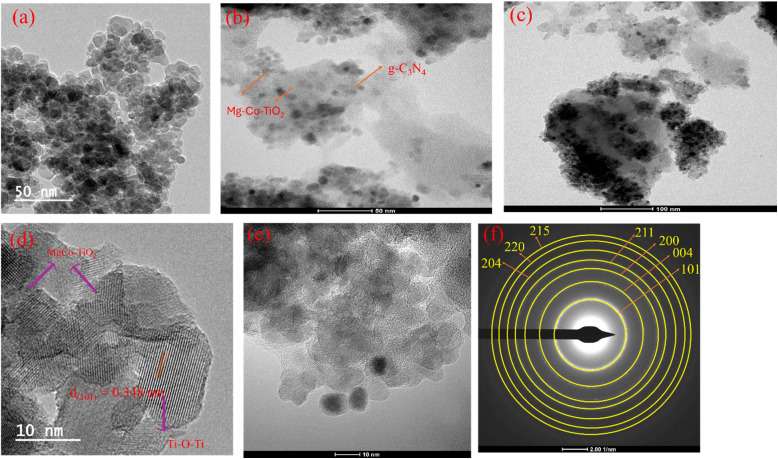
TEM images of TiO_2_ (a) and MgCo-TiO_2_/g-C_3_N_4_ (b and c), HRTEM images of TiO_2_ (d) and MgCo-TiO_2_/g-C_3_N_4_ (e) and SAED pattern of MgCo-TiO_2_/g-C_3_N_4_ (f).

The TEM analysis (Fig. 5a) revealed that the MgCo-TiO_2_ nanoparticles have a predominantly spherical morphology with slight aggregation. As shown in Fig. 5b and c, these nanoparticles are uniformly coated onto the agglomerated g-C_3_N_4_ sheets, where the black spherical structures correspond to MgCo-TiO_2_ and the gray nanosheet background represents g-C_3_N_4_.^42^ The HRTEM image (Fig. 5d) displayed a clear lattice fringe with a *d*-spacing of 0.35 nm, corresponding to the (101) plane of anatase TiO_2_, as measured using the ImageJ software. Although Mg and Co doping did not alter the crystal structure or phase of TiO_2_, it improved the nanoparticle dispersion and reduced the band gap. In contrast, the MgCo-TiO_2_ coated g-C_3_N_4_ sheets (Fig. 5e) appeared with a disordered interface region, which made the fringes indistinct, indicating the formation of a heterostructure. The SAED pattern of MgCo-TiO_2_/g-C_3_N_4_ (Fig. 5f) showed well-defined rings corresponding to the tetragonal anatase phase of TiO_2_, while g-C_3_N_4_ exhibited no distinct rings due to its amorphous or semi-crystalline nature. Overall, these results confirm the successful coating of the MgCo-TiO_2_ nanoparticles onto layered g-C_3_N_4_, forming heterostructure nanocomposites.

#### 3.1.8. FESEM

**Fig. 6:**
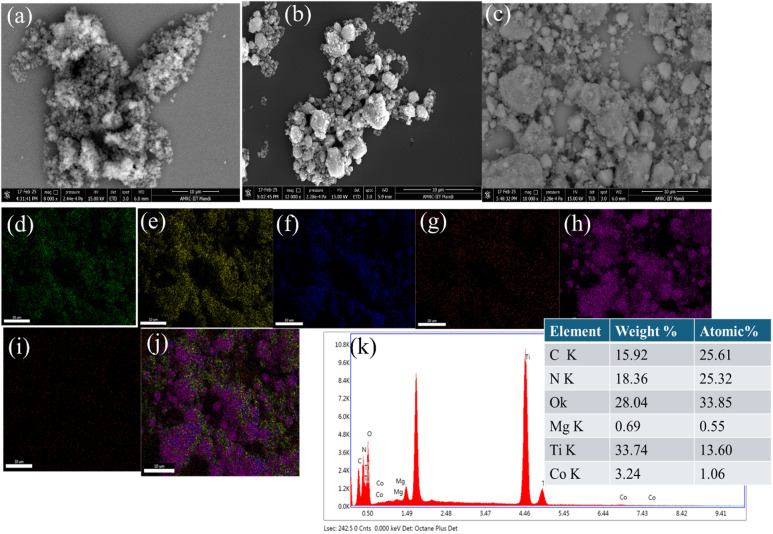
FESEM images of g-C_3_N_4_ (a), MgCo-TiO_2_ (b) and MgCo-TiO_2_/g-C_3_N_4_ (c), elemental distribution mapping of C (d), N (e), O (f), Mg (g), Ti (h), and Co(i), combined elemental mapping in MgCo-TiO_2_/g-C_3_N_4_ (j) and EDX spectrum of MgCo-TiO_2_/g-C_3_N_4_ (k).

As shown in Fig. 6, the surface morphologies of the g-C_3_N_4_, TiO_2_/g-C_3_N_4_, and MgCo-TiO_2_/g-C_3_N_4_ synthesized nanomaterials were examined using SEM analysis. The g-C_3_N_4_ polymer (Fig. 6a) exhibited a layered nanosheet structure resembling loosely stacked graphite-like layers with agglomerated sheets. The MgCo-TiO_2_ nanoparticles (Fig. 6b) displayed a nearly spherical morphology with slight aggregation. The heterostructure composites (Fig. 6c) showed a coupled structure, where spherical MgCo-TiO_2_ nanoparticles were uniformly coated onto the g-C_3_N_4_ nanosheets, confirming their successful interfacial contact. EDX analysis and elemental mapping (Fig. 6d–i) verified the presence of C, N, O, Mg, Ti, and Co, with a uniform distribution across the composite. The combined elemental map (Fig. 6j) further demonstrated that Ti, Mg, and Co signals were concentrated on the outer surface, while the C and N signals overlapped with them, confirming the successful coating of Mg–Co-doped TiO_2_ onto g-C_3_N_4_ and the formation of heterostructure nanocomposites.

#### 3.1.9. FT-IR characterization

The functional groups and chemical bonding characteristics of g-C_3_N_4_, TiO_2_, and the MgCo-TiO_2_/g-C_3_N_4_ heterostructures were investigated using Fourier transform infrared spectroscopy (FT-IR) (Fig. 1d). In the spectrum of pure TiO_2_, the pronounced absorption band at 506 cm^−1^ corresponds to the Ti–O–Ti stretching vibration of the TiO_2_ lattice. In the case of pristine g-C_3_N_4_, the characteristic absorption band at 1641 cm^−1^ is attributed to C=N stretching vibrations, while the peaks at 1411 cm^−1^ and 1355 cm^−1^ are associated with the aromatic C–N stretching modes.^43,44^ Additionally, the band observed at 804 cm^−1^ is assigned to the *s*-triazine ring breathing mode, a structural feature of g-C_3_N_4_. Upon the incorporation of TiO_2_ and MgCo-TiO_2_ onto the surface of g-C_3_N_4_, the intensity of this band decreased and became broader, suggesting successful surface coating and the formation of metal–nitrogen (M–N; M = Ti, Mg, and Co) interactions between the doped TiO_2_ and g-C_3_N_4_. Furthermore, the broad absorption band in the range of 3060–3540 cm^−1^ corresponds to the N–H stretching vibrations of amine groups and O–H stretching from surface-adsorbed hydroxyl species, confirming the presence of hydrogen-bonded surface functionalities.

### 3.2. Electrochemical characterization of MgCo-TiO_2/_g-C_3_N_4_

**Fig. 7:**
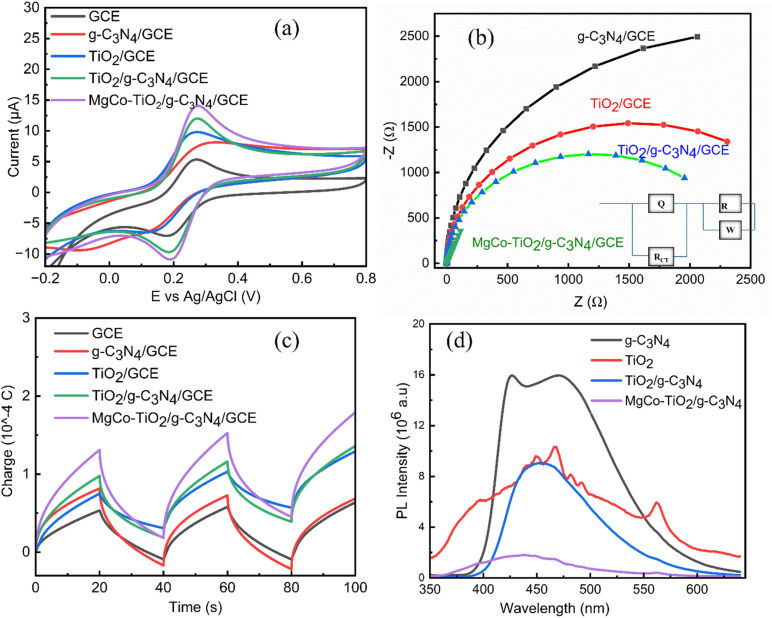
CV curves (a), EIS Nyquist plots (b), chronocoulometric curves (c) and PL spectra of TiO_2_, g-C_3_N_4_, MgCo-TiO_2,_ TiO_2_/g-C_3_N_4_ and MgCo-TiO_2_/g-C_3_N_4_ (d).

The electrochemical properties of the samples were evaluated using various techniques including electrochemical impedance spectroscopy (EIS), cyclic voltammetry (CV), and chronocoulometry. Each catalyst, g-C_3_N_4_, TiO_2_, TiO_2_/g-C_3_N_4_ and MgCo-TiO_2_/g-C_3_N_4_, was compared and investigated using the known redox behavior of 2 mM K_3_[Fe(CN)_6_] electrolyte solution with 0.1 M KCl as the supporting electrolyte. As shown in Fig. 7a, the cyclic voltammetry characterization of all the synthesized materials g-C_3_N_4_, TiO_2_, TiO_2_/g-C_3_N_4_, and MgCo-TiO_2_/g-C_3_N_4_ exhibited well-defined and reversible redox peaks. The oxidation and reduction currents corresponding to the Fe^2+^/Fe^3+^ redox couple in the electrolyte increased progressively in the following order: bare GCE < g-C_3_N_4_/GCE < TiO_2_/GCE < TiO_2_/g-C_3_N_4_/GCE < MgCo-TiO_2_/g-C_3_N_4_/GCE. The obtained anodic current response for each electrode GCE, g-C_3_N_4_/GCE, TiO_2_/GCE, TiO_2_/g-C_3_N_4_/GCE, and MgCo-TiO_2_/g-C_3_N_4_/GCE is 5.56, 8.21, 9.79, 12.05 and 14.25 µA, respectively. The active surface area of the electrodes was calculated using the Randles–Sevcik equation, as follows:
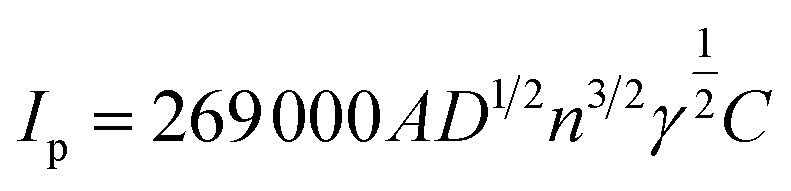
where *I*_p_ is the peak current, *A* is the electrochemical active surface area, *D* is the diffusion coefficient, which is 6.7 × 10^−6^ cm^2^ s^−1^, *n* is number of electrons, which is 1, *C* is the concentration of electrolyte, *i.e.*, 2 × 10^−6^ mol cm^−3^ for the given electrolyte, and *γ* is the scan rate, *i.e.*, 0.05 V s^−1^. The obtained electrochemical active surface area for GCE, g-C_3_N_4_/GCE, TiO_2_/GCE, TiO_2_/g-C_3_N_4_/GCE, MgCo-TiO_2_/g-C_3_N_4_/GCE is 0.018 cm^2^, 0.026 cm^2^, 0.031 cm^2^, 0.039 cm^2^ and 0.046 cm^2^, respectively. These results indicate that the electrochemical conductivity improves with g-C_3_N_4_ surface modification, particularly with TiO_2_ and Mg–Co co-doped TiO_2_. This enhancement is attributed to the increased surface area of g-C_3_N_4_, which promotes greater ion adsorption, and the presence of defect-trap dopants, suppressing electron–hole recombination.

As shown in Fig. 7b, the electrochemical conductivity performance of all the synthesized catalysts was evaluated and compared using EIS. The Nyquist plots of the prepared catalysts display two characteristic regions, a semicircular arc in the high-frequency range, corresponding to charge-transfer processes, and a straight line at lower frequencies, associated with ion diffusion. The charge-transfer resistance (*R*_ct_) for each catalyst-coated glassy carbon electrode was estimated from the intercept of the semicircle with the real (*Z*′) axis. Among the tested materials, g-C_3_N_4_, TiO_2_, TiO_2_/g-C_3_N_4_, and MgCo-TiO_2_/g-C_3_N_4_, MgCo-TiO_2_/g-C_3_N_4_ heterostructure exhibited the smallest semicircle diameter, reflecting a substantial decrease in *R*_ct_ from 1123 Ω (g-C_3_N_4_) to 129.38 Ω. This significant reduction in resistance confirms that the MgCo-TiO_2_/g-C_3_N_4_ heterostructure possesses the most efficient charge transfer and superior electrochemical conductivity among the evaluated catalysts.

The quantity of ions absorbed by the catalyst on the electrode surface was evaluated through potential step experiments using the chronocoulometric technique (Fig. 7c). The charge associated with the electrolyte for each catalyst-coated electrode was determined from its charge *versus* time plots.^45^ During the first 60 s of the anodic step, the charge (*q*) associated with Fe^2+^/Fe^3+^ adsorption was measured for each catalyst-modified electrode. The *q* value for GCE, g-C_3_N_4_/GCE, TiO_2_/GCE, TiO_2_/g-C_3_N_4_/GCE and MgCo-TiO_2_/g-C_3_N_4_/GCE was 0.58 × 10^−4^ C, 0.725 × 10^−4^ C, 1.035 × 10^−4^ C, 1.155 × 10^−4^ C and 1.53 × 10^−4^ C, respectively. Among them, the MgCo-TiO_2_/g-C_3_N_4_ catalyst exhibited the highest charge, reflecting its greater ion uptake due to its higher porosity and surface area. Therefore, the MgCo-TiO_2_/g-C_3_N_4_ catalyst-coated GCE is suitable for electrochemical sensor applications due to its improved electrocatalytic performance.

### 3.3. Electrochemical detection of 2,4-DNPH using MgCo-TiO_2_/g-C_3_N_4_/GCE

**Fig. 8:**
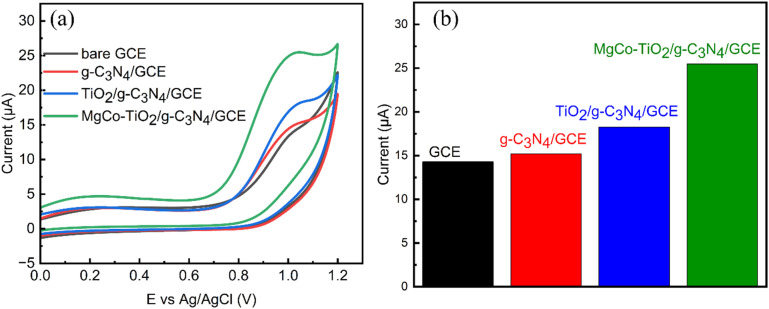
CV curves (a), and bar diagram for GCE, TiO_2_/g-C_3_N_4_/GCE and MgCo-TiO_2_/g-C_3_N_4_/GCE (b).

**Scheme 3:**
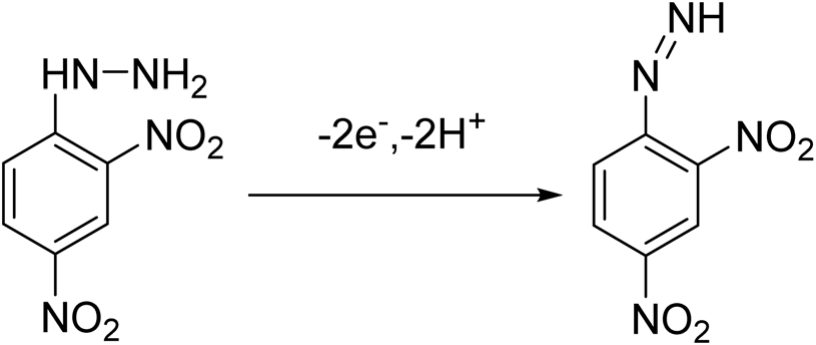
Electrochemical oxidation of 2,4-dinitrophenylhydrazin to 2,4-dinitrophenyl diazene.

2,4-Dinitrophenylhydrazine (2,4-DNPH) contains a hydrazine (–NH–NH_2_) functional group, which is electrochemically active due to its electron-rich nature. However, the presence of strongly electron-withdrawing nitro groups at the 2 and 4 positions of its aromatic ring affects the oxidation process, causing the –NH–NH_2_ group to undergo electrochemical oxidation at a relatively higher positive potential. As shown in Fig. 8a, the electrochemical oxidation of 2,4-DNPH was studied using cyclic voltammetry at a concentration of 0.4 µM in a solution with pH 3. The oxidation peak was observed at a potential of 1.02 V using a glassy carbon electrode as the working electrode. At this higher potential, the –NH–NH_2_ group oxidized to aryl diazene (Ar–N=NH) at the nitrogen center (Scheme 3),^46^ rather than the reduction of the nitro groups, which typically takes place at lower negative potentials. To evaluate and compare the electroanalytical responses toward 2,4-DNPH detection, different modified electrodes were used, including bare GCE, g-C_3_N_4_/GCE, TiO_2_/g-C_3_N_4_/GCE and MgCo-TiO_2_/g-C_3_N_4_/GCE. The corresponding oxidation current response values obtained were 14.29, 15.19, 18.26, and 25.47 µA, respectively. Graphitic carbon nitride contains electron-rich nitrogen groups, which can chemically interact with the functional groups of 2,4-DNPH, offering potential for electrochemical interactions. However, due to the inherently low surface area and poor electrical conductivity of g-C_3_N_4_, its electroanalytical response was not significantly enhanced. Thus, to improve this, TiO_2_ nanoparticles were coated onto the surface of g-C_3_N_4_, forming a TiO_2_/g-C_3_N_4_ heterostructure catalyst. This modification improved the electrochemical communication between the electrolyte solution and the glassy carbon electrode by increasing both the surface area and conductivity. Nevertheless, the wide bandgap of TiO_2_ leads to electron–hole recombination, which limits the further enhancement of the analytical response. Thus, to overcome this limitation and achieve a better electrochemical performance, a two metal-doping approach was employed, where Mg and Co were co-doped into TiO_2_, and subsequently coated onto g-C_3_N_4_. As shown in the bar graph in Fig. 8b, MgCo-TiO_2_/g-C_3_N_4_/GCE exhibited the highest electroanalytical response toward 2,4-DNPH, which is attributed to the synergistic effects of the heterostructure nanocomposite. Therefore, MgCo-TiO_2_/g-C_3_N_4_/GCE was selected for further optimization and applied in the electrochemical sensing of 2,4-DNPH.

#### 3.3.1. Effect of pH

**Fig. 9:**
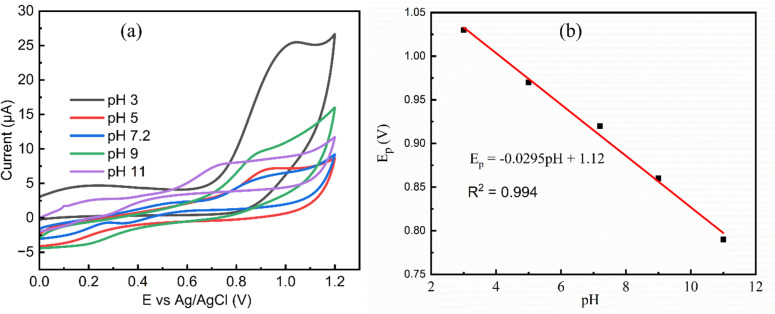
CV curves of MgCo-TiO_2_/g-C_3_N_4_/GCE under a constant concentration of 0.4 µM 2,4-DNPH over a range of pH values (a) and linear relationship between its potential peak and pH at a scan rate of 30 mV s^−1^ (b).

The solution pH significantly influences the electrochemical oxidation of the hydrazine functional group in 2,4-DNPH due to protonation and deprotonation processes. The electrochemical sensing performance of MgCo-TiO_2_/g-C_3_N_4_/GCE was evaluated using CV in a the same 0.4 µM 2,4-DNPH concentration over a range of pH values. As illustrated in Fig. 9a, the oxidation peak potential shifts to less positive values with an increase in pH. This behavior can be attributed to the reduced availability of H^+^ ions, which are essential for protonating the –NH–NH_2_ group during the oxidation process of 2,4-DNPH at the MgCo-TiO_2_/g-C_3_N_4_/GCE surface. Fig. 9b further demonstrates a linear correlation between the peak potential and pH, which can be expressed as follows:*E*_p_ (V) = −0.0295 pH + 1.12

The slope of 0.0295 V pH^−1^ is approximately half of the theoretical Nernstian value (0.059 V pH^−1^), indicating a proton-to-electron ratio of 1 : 2. During the oxidation process, two hydrogen atoms are removed (corresponding to the release of two protons, 2H^+^) to form Ar–N=NH. However, the observed half-Nernstian slope of 0.0295 V pH^−1^ indicates that only one proton is directly involved in the potential-determining step. The second proton is likely released through a subsequent, rapid radical rearrangement step that facilitates the stabilization of the intermediate species. This behavior is a characteristic feature of the electrochemical oxidation of 2,4-DNPH, where the hydrazine nitrogen serves as the electron-active center. The MgCo-TiO_2_/g-C_3_N_4_ heterostructure catalyst-modified GCE exhibits an oxidation current over a wide pH range, including neutral pH, which is particularly advantageous for the electrochemical detection of hazardous organic compounds in real environmental samples, given that most of these samples typically have a neutral pH.^14^ However, to enhance the sensitivity and achieve a sharper oxidation peak current with a highest intensity at lower concentrations of 2,4-DNPH, pH 3 was selected as the optimal working condition in this study.

#### 3.3.2. Effect of scan rate

**Fig. 10:**
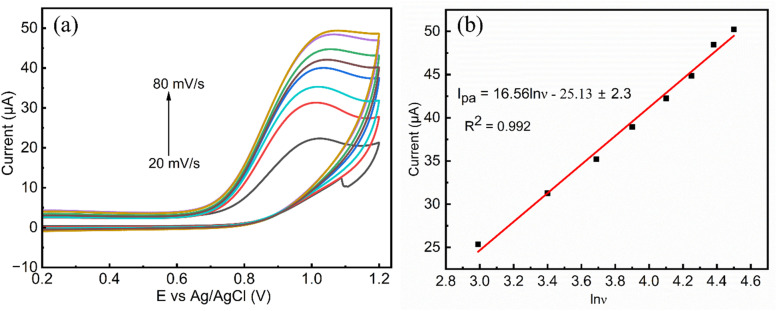
(a) MgCo-TiO_2_/g-C_3_N_4_/GCE CV curves at different scan rates (20, 30, 40,50, 60, 70, 80, and 90 mV s^−1^) with 0.4 µM 2,4-DNPH solution in 0.1 M PBS (pH 3) and (b) plot of linear regression redox peak current *vs.* square root of the scan rate.

The scan rate plays a crucial role in determining the electrochemical behavior during electrochemical sensing. Most organic compounds display sharp and well-defined peaks at lower scan rates, whereas at higher scan rates, their peaks tend to become broader when analyzed using the cyclic voltammetry technique. As illustrated in Fig. 10a, the effect of scan rate was investigated for the MgCo-TiO_2_/g-C_3_N_4_-modified electrode as an electrochemical sensor for 0.4 µM 2,4-DNPH solution, at a potential in the range of 0 to 1.2 V at various scan rates (20, 30, 40, 50, 60, 70, 80, and 90 mV s^−1^). As shown in Fig. 10b, with an increase in the scan rate, the anodic peak current increased linearly, indicating enhanced conductivity by the catalyst and diffusion-controlled behavior. A clear and defined peak was observed at 30 mV s^−1^ and this lower scan rate is recommended for the electrochemical detection of organic compounds, and therefore selected as the optimal scan rate for the MgCo-TiO_2_/g-C_3_N_4_/GCE sensor for 2,4-DNPH detection.

#### 3.3.3. Effect of 2,4-DNPH concentration

**Fig. 11:**
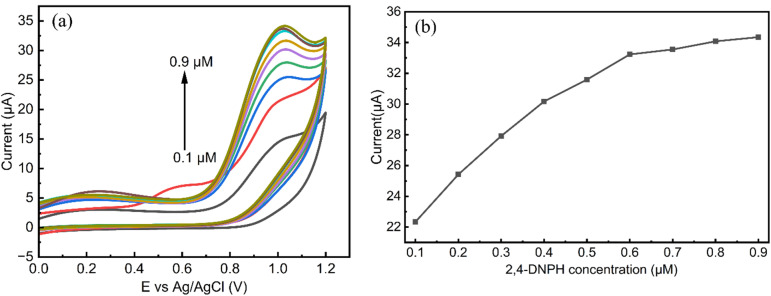
CV curves for different 2,4-DNPH concentrations in 0.1 M PBS and pH 3 at a scan rate of 30 mV s^−1^ (a) and linear calibration of oxidation peak current *vs.* 2,4-DNPH concentration (b).

The influence of 2,4-DNPH concentration on the electrochemical performance of the MgCo-TiO_2_/g-C_3_N_4_ catalyst-modified GCE was examined using cyclic voltammetry at different concentrations (0.1, 0.2, 0.3, 0.4, 0.5, 0.6, 0.7, 0.8 and 0.9 µM) in a pH 3 solution, within the potential range of 0 to 1.2 V at a scan rate of 30 mV s^−1^, as presented in Fig. 11a. As the concentration of 2,4-DNPH increased, the oxidation peak current showed a linear increase from 0.1 to 0.6 µM (Fig. 11b), indicating that the MgCo-TiO_2_/g-C_3_N_4_ catalyst possesses high porosity and more active sites, which enhance the electrocatalytic interaction between 2,4-DNPH and the GCE surface. However, at concentrations of 0.7 µM to 0.9 µM, the oxidation peak current plateaued, suggesting that the effective working range of the developed sensor for 2,4-DNPH detection using the cyclic voltammetry technique is between 0.1 and 0.6 µM.

#### 3.3.4. Electrochemical detection of 2,4-DNPH using LSV

**Fig. 12:**
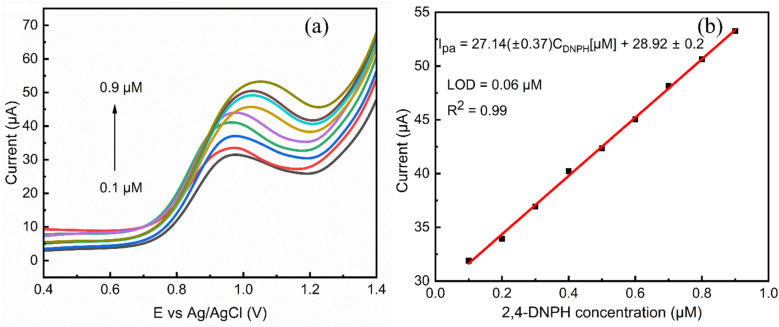
(a) LSV voltammogram for different 2,4-DNPH concentrations (0.1, 0.2, 0.3, 0.4, 0.5, 0.6, 0.7, 0.8, and 0.9 µM) in 0.1 M PBS at pH 3 with a corrected current base line and (b) linear calibration of oxidation peak current *vs.* 2,4-DNPH concentration.

MgCo-TiO_2_/g-C_3_N_4_/GCE was employed as an electrochemical sensor for the detection of 2,4-DNPH using the linear sweep voltammetry (LSV) technique, which is more sensitive compared to CV, under the optimized conditions with varying analyte concentrations. As shown in Fig. 12a, the oxidative current of 2,4-DNPH increases linearly with an increase in concentration from 0.1 to 0.9 µM, which can be attributed to the high surface area and abundant reactive sites of the catalyst on the GCE surface. Consequently, the electroanalytical current response also increased with an increase in the analyte concentration. The calibration plot of LSV current *versus* 2,4-DNPH concentration (Fig. 12b) demonstrates a strong linear relationship in the range of 0.1 to 0.9 µM, represented by the equation *I*_pa_ = 27.14(±0.37)*C*_DNPH_ [µM] + 28.92 ± 0.2, with a correlation coefficient (*R*^2^) of 0.99. The sensitivity was determined from the slope of the calibration plot, normalized by the electrochemically active surface area (ECSA) of the modified electrode (sensitivity = slope/ECSA). The ECSA was evaluated from the cyclic voltammetry data using the Randles–Ševčík equation (0.046 cm^2^), yielding an impressive sensitivity of 589.13 µA µM^−1^ cm^−2^, and highlighting the excellent electrochemical performance of the electrode. The LOD was calculated using the formula LOD = 3*σ*/*m*, where *σ* represents the standard deviation of the blank solution of 9 measurements and m represents is the slope of the calibration curve. The calculated detection limit was 0.06 µM. The limit of detection and linear range for 2,4-DNPH detection in this work is compared with that reported in previous studies in Table 5.

**Table 5:** Comparison of the electrocatalytic and photocatalytic performance of different catalysts for the detection of 2,4-DNPH

Electrocatalytic performance comparison					
S. no.	Sensor	Technique	Linear range (µM)	LOD (µM)	Ref.
1	Zn-MOF sensor	Fluorescent	0.1 to 500	0.01	13
2	Cu(ii) at GCE	EIS	—	0.04	46
3	P-/*p*ABA-MnO_2_	LSV	0.5 to 90	0.08	2
4	(P_8_W4_8_/PDDA)_7_	Amperometry	1 to 700	0.012	14
5	MgCo-TiO_2_/g-C_3_N_4_/GCE	LSV	0.1 to 0.9	0.06	This work

#### 3.3.5. Interference study of MgCo-TiO_2_/g-C_3_N_4_ catalyst

The selectivity of the developed MgCo-TiO_2_/g-C_3_N_4_-modified GCE sensor was evaluated using Na^+^ and Pb^2+^ ions as potential interferents, given that the nitrogen sites in g-C_3_N_4_ can interact with protons and metal cations. In this study, a solution containing 0.4 µM 2,4-DNPH, 0.4 µM NaNO_3_, and 0.4 µM Pb(NO_3_)_2_ was prepared, and its response was measured using LSV under the optimized conditions. As shown in Fig. S2a, the presence of Na^+^ and Pb^2+^ ions resulted in only an 8% decrease in the maximum oxidation current of 2,4-DNPH. Additionally, because nitrogen in g-C_3_N_4_ can also interact with hydroxyl groups, the interference effect of 4-nitrophenol was examined by adding an equal concentration of 4-nitrophenol to the 0.4 µM 2,4-DNPH solution. The LSV results (Fig. S2b) showed that the maximum current of 2,4-DNPH decreased by only 3.08% in the presence of 4-nitrophenol. Furthermore, the interference from nitrobenzene was studied in 0.4 µM 2,4-DNPH and 0.4 µM nitrobenzene solution. After the addition of nitrobenzene, the maximum LSV current response of DNPH decreased by 4.96% due to the electron-withdrawing nitro group (Fig. S3). These findings demonstrate that the MgCo-TiO_2_/g-C_3_N_4_/GCE sensor exhibits excellent selectivity for the detection of 2,4-DNPH.

#### 3.3.6. Reproducibility, repeatability and long-term stability study of MgCo-TiO_2_/g-C_3_N_4_ catalyst

After modifying the GCE surface with the MgCo-TiO_2_/g-C_3_N_4_ catalyst, the repeatability and long-term stability of the electrode were assessed using a 2,4-DNPH solution in an acidic medium *via* the LSV technique. The repeatability of the MgCo-TiO_2_/g-C_3_N_4_/GCE sensor for detecting 0.4 µM 2,4-DNPH was evaluated under the optimized conditions by performing four consecutive measurements with the same electrode (Fig. S4a). The relative standard deviation (RSD) for these four assays was 1.38%, indicating the high precision and repeatability of the developed sensor toward 2,4-DNPH detection. The reproducibility of the MgCo-TiO_2_/g-C_3_N_4_/GCE sensor for detecting 2,4-DNPH using the LSV technique was evaluated by measuring the responses of three independently fabricated MgCo-TiO_2_/g-C_3_N_4_/GCE sensors under the same conditions. The calculated RSD% was found to be 3.57%.

The long-term stability of the fabricated MgCo-TiO_2_/g-C_3_N_4_/GCE sensors for the electrochemical detection of 2,4-DNPH was evaluated using the LSV technique (Fig. S4b). Measurements were taken on the first day and again after storing the electrodes in air at room temperature for five weeks. The results showed a 2.37% decrease in the initial oxidation current, indicating that the catalyst coating remained firmly attached to the GCE surface, likely due to the strong carbon–carbon bonding between the GCE and g-C_3_N_4_ in the catalyst.

#### 3.3.7. Real sample studies

The practical applicability of the developed electrochemical sensor toward 2,4-DNPH detection was evaluated using organic laboratory effluents collected from the Department of Chemistry, Andhra University, India. The wastewater samples were initially centrifuged at 2000 rpm, and the resulting supernatant was filtered through Whatman filter paper prior to analysis. Because the saturated aqueous DNPH suspension was measured to be a weak acid (pH ≈ 5.83), it exhibits nearly neutral charge, and is poorly soluble in water, and thus the electrochemical measurements were performed in 0.2 M aqueous HCl solution (pH 3) to ensure adequate solubility and charge mobility. The filtered samples were analyzed in triplicate (*n* = 3), and the native 2,4-DNPH concentration in the matrix was quantified to be 0.17 µM using spectrophotometric analysis. The developed electrochemical sensor detected 0.15 µM concentration in the real sample. The recovery studies were performed *via* the standard addition method by spiking the real samples with 0.3, 0.4, and 0.5 µM 2,4-DNPH standards. The calculated recovery percentages (*R*%) ranged from 95.74% to 101.49%, confirming the high accuracy and reliability of the proposed sensor for real sample analysis (Table 6).

**Table 6:** Determination of 2,4-DNPH in wastewater effluent and recovery test for MgCo-TiO_2_/g-C_3_N_4_/GCE sensor

Native 2,4-DNPH (µM)	Replicates (*n*)	Added (µM)	Obtained by sensor (µM)	RSD (%)	Recovery (%)
0.17	3	0	0.15	2.25	—
	3	0.3	0.45	2.48	95.74
	3	0.4	0.56	3.35	98.24
	3	0.5	0.68	2.57	101.49

## 4. Conclusion

In this work, we successfully enhanced the conductivity, specific surface area, and surface properties of g-C_3_N_4_ nanosheets through surface modification with TiO_2_ nanoparticles to form TiO_2_/g-C_3_N_4_ heterostructure nanocomposites. However, the large band gap of TiO_2_ (3.2 eV) promotes rapid electron–hole recombination, thereby reducing the conductivity of the heterostructure. Thus, to overcome this limitation, TiO_2_ nanoparticles were doped with Mg and Co prior to coupling with g-C_3_N_4_. Co-doping reduced their band gap to 2.58 eV, decreased their crystalline particle size to 6.28 nm, and introduced new electronic states, where Co contributed transitional d-orbital states, while Mg generated oxygen vacancy states. The integration of these improved TiO_2_ nanoparticles with g-C_3_N_4_ nanosheets produced MgCo-TiO_2_/g-C_3_N_4_ heterostructure nanocomposites with enhanced conductivity and enlarged surface area (81.61 m^2^ g^−1^). When coated on a GCE, the MgCo-TiO_2_/g-C_3_N_4_ catalyst exhibited an excellent performance for the electrochemical sensing of 2,4-DNPH, demonstrating high selectivity, sensitivity, stability, reproducibility, and a low detection limit of 0.06 µM. Furthermore, the developed sensing platform holds great promise for monitoring other hazardous organic pollutants in environmental systems.

## Author contributions

Samuel Chufamo Jikamo (first author): conceived and designed the study, conducted the primary research, analyzed the data, and prepared the main manuscript. He was primarily responsible for drafting, structuring, and organizing the content of the manuscript. Prof. T. Sivarao (corresponding author): critically revised and edited the manuscript for intellectual content, grammar, structure, and clarity. Prof. P. Shyamala (co-author): conducted a thorough review of the manuscript and offered constructive feedback, which greatly improved its coherence over several rounds of revision. Singupilla Sai Supriya (co-author): thorough meticulous review and constructive input. Sandhya Rani Nayak (co-author): thoroughly reviewed the manuscript and provided insightful minor revisions. Nageswararao Kadiyala (co-author): reviewing and refining the manuscript in terms of technical formatting and content alignment with visual elements. Winni Teja Dokka (co-author): reviewed the manuscript. M. Ravichandra: instrumental characterization. M. V Kishore: reviewed the manuscript.

## Conflicts of interest

The authors declare no competing interests.

## Supplementary Material

RA-015-D5RA07106B-s001

## Data Availability

All data generated or analyzed during this study are included in the manuscript. Supplementary information (SI) is available. See DOI: https://doi.org/10.1039/d5ra07106b.
