# Correction: Highly sensitive electrochemical detection of hazardous 2,4-dinitrophenylhydrazine using MgCo-TiO_2_/g-C_3_N_4_ heterostructure nanocomposites

**DOI:** 10.1039/d6ra90032a

**Published:** 2026-04-28

**Authors:** Samuel Chufamo Jikamo, T. Siva Rao, P. Shyamala, Singupilla Sai Supriya, Sandhya Rani Nayak, Nageswararao Kadiyala, Winni Teja Dokka, M. Ravi Chandra, M. V. Kishore

**Affiliations:** a Dept of Chemistry, Andhra University Visakhapatnam 530003 India sivaraoau@gmail.com; b Dept of Chemistry, College of Natural and Computational Sciences, Wolaiat Sodo University Wolaita Sodo Ethiopia; c Dept of Chemistry, Dr V S Krishna Govt. Degree College (A) Visakhapatnam 530013 India; d Dept of Chemistry, Govt. Degree College Chintalapudi West Godavari 534460 India; e Department of Chemistry, Indian Institute of Technology Patna Bihta, Patna Bihar 801106 India

## Abstract

Correction for ‘Highly sensitive electrochemical detection of hazardous 2,4-dinitrophenylhydrazine using MgCo-TiO_2_/g-C_3_N_4_ heterostructure nanocomposites’ by Samuel Chufamo Jikamo *et al.*, *RSC Adv.*, 2025, **15**, 45855–45873, https://doi.org/10.1039/D5RA07106B

The authors regret that the name of one of the authors was incorrect in the original manuscript. In addition, the authors regret that one of the affiliations (affiliation *e*) was incorrectly shown in the original manuscript. The corrected list of authors and affiliations are as shown here.

The Royal Society of Chemistry apologises for these errors and any consequent inconvenience to authors and readers.

