# Supplementary material for: Highly sensitive electrochemical detection of hazardous 2,4-dinitrophenylhydrazine using MgCo-TiO2/g-C3N4 heterostructure nanocomposites
Source: RSC Adv. 2025 Nov 24;15(54):45855–73. doi: 10.1039/d5ra07106b (PMC12642856; doi:10.1039/d5ra07106b)
Supplement: RA-015-D5RA07106B-s001 [file RA-015-D5RA07106B-s001.pdf]

Supplementary Information (SI) for RSC Advances.
This journal is © The Royal Society of Chemistry 2025

Highly sensitive electrochemical detection of hazardous 2,4-dinitrophenylhydrazine using $MgCo\text{-}TiO_2/g\text{-}C_3N_4$ heterostructure nanocomposites

Samuel Chufamo Jikamo [a,b], T. Siva Rao* [a] P. Shyamala [a], Singupilla Sai Supriya [a], Sandhya Rani Nayak [a], Nageswararao Kadiyala [a,c], Winni Teja Dokka [a,d] M. Ravichandra[e], M.V Kishore[a]

[a] Dept of Chemistry, Andhra University, Visakhapatnam, India-530003.

[b] Dept of Chemistry, College of Natural and Computational sciences, Wolaiat Sodo University, Wolaita Sodo, Ethiopia.

[c] Dept of Chemistry, Dr. V S Krishna Govt. Degree College(A), Visakhapatnam, India-530013.

[d] Dept of Chemistry, Govt. Degree College Chintalpudi, West Godavari, India-534460.

[e] C4-101, Staff Quarters, Indian Institute of Technology Patna, Bihta, Bihar, India-801106.

*Corresponding author: T.Siva Rao

Corresponding author email: sivaraoau@gmail.com

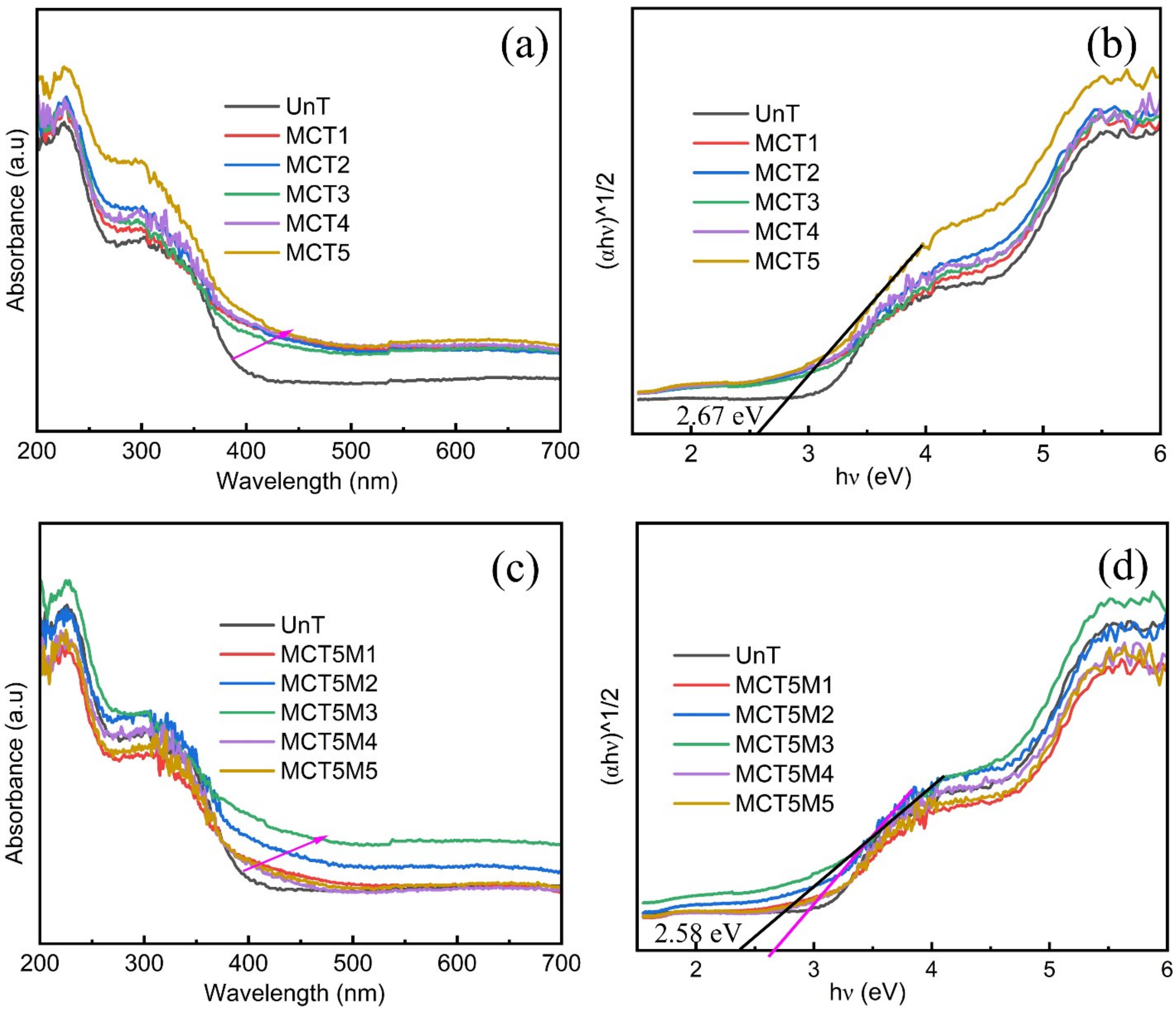

Fig. S1 UV-Vis DRS spectra for sol gel synthesized absorbance (**a**) and along with the square root of Kubelka–Munk functions (**b**) and microwave assisted sol gel synthesized materials (**c**) and along with the square root of Kubelka–Munk functions (**d**).

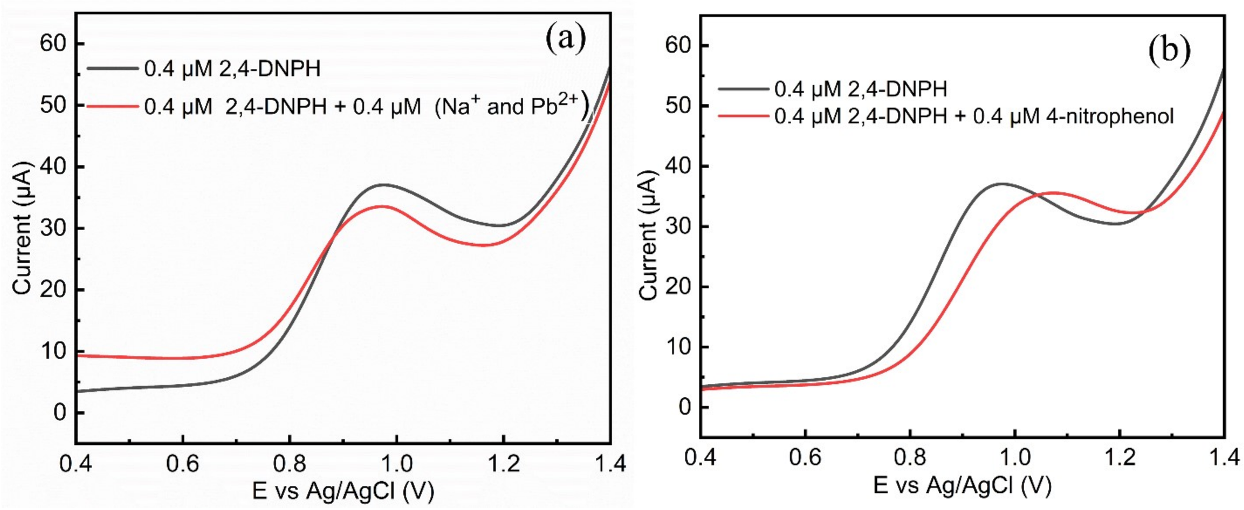

Fig. S2 LSV Voltammogram metals ions (Na^{+} and Pb^{2+}) (**a**) and 4-nitrophenol(**b**) interferences in 0.4 µM 2,4-DNPH (pH 3) at scan 30 mV/s scan rate by MgCo-TiO_2/g-C_3N_4/GCE sensor.

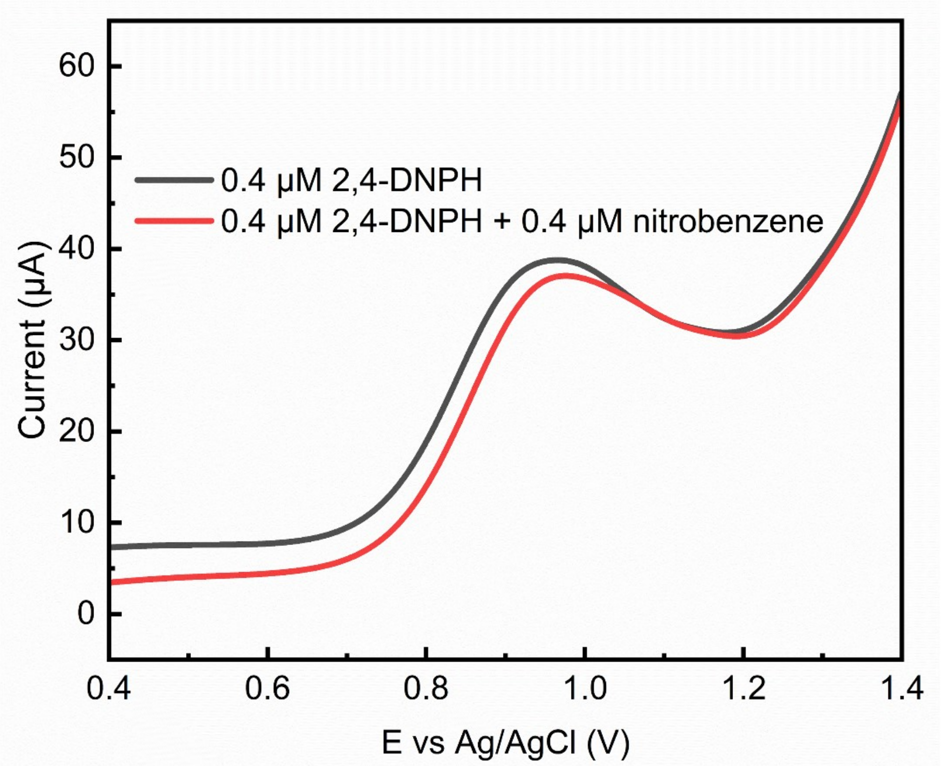

Fig.S3 LSV voltammogram 4-nitrobenzene interferences in 0.4 µM 2,4-DNPH (pH 3) at scan 30 mV/s scan rate by MgCo-TiO_2/g-C_3N_4/GCE sensor.

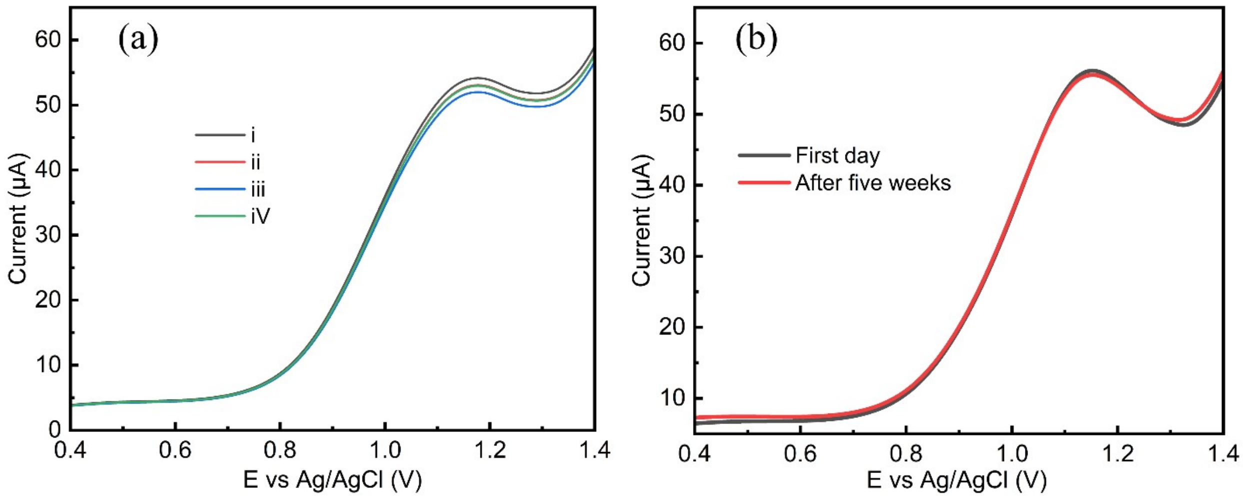

Fig.S4 LSV voltammogram of four consecutive measurements (**a**) and stability(**b**) in 0.4 µM 2,4-DNPH (pH 3) at scan 30 mV/s scan rate by MgCo-TiO_2/g-C_3N_4/GCE sensor.
